# Beyond flirtation: social sexual behavior and employee innovation — the moderating effects of gender and social sexual behavior frequency

**DOI:** 10.3389/fpsyg.2025.1537617

**Published:** 2025-09-08

**Authors:** Eunmi Jang

**Affiliations:** Part of Creative Thinking & Innovation, Baird Liberal Arts College, Soongsil University, Seoul, Republic of Korea

**Keywords:** social sexual behavior, psychological social resources, innovative behavior, gender, social sexual behavior frequency, moderating effect

## Abstract

This study examines how supervisors’ social sexual behavior (SSB) affects subordinates’ psychological social resources and innovative behavior, focusing on the moderating effects of gender and SSB frequency. Survey data from 212 employees in South Korean private sector organizations indicate that positive perceptions of supervisors’ SSB enhance subordinates’ psychological social resources, which in turn promote innovative behavior. Gender moderates the relationship between SSB and psychological social resources, with female employees experiencing stronger positive effects than males. However, high frequency of SSB weakens this positive effect, suggesting an optimal level of such behavior. These findings advance the literature on workplace SSB by demonstrating its potential to foster innovation through psychological resources while highlighting individual and contextual differences. Implications for organizations include fostering innovation-supportive environments that respect appropriate interpersonal boundaries.

## 1 Introduction

Organizations, as human collectives, are characterized by various forms of interaction, with informal interactions occurring beneath formal role relationships playing a crucial part (Homans, 1950). Among these various interactions, sexuality-related behaviors are commonly observed phenomena in organizational settings (Berdahl and Aquino, 2009; Gutek, 1985). Human sexuality is not merely an instinct to be suppressed, but rather a vital energy that can contribute to positive organizational dynamics. Indeed, Williams et al. (1999) demonstrated that sexuality-related dynamics in organizational mentoring relationships can function constructively. However, in South Korea, where traditional Confucian culture remains influential, sexuality-related behaviors are often treated as taboo or inappropriate (Park and Kim, 2019). This cultural context is reflected in organizational behavior research, where sexuality-related variables, except for workplace romance (e.g., Pierce, 1998; Pierce and Aguinis, 2009; Quinn, 1977), have predominantly been examined through negative lenses such as sexual harassment (Raver and Nishii, 2010) or workplace incivility (Lim and Cortina, 2005).

Contrary to this predominantly negative perspective, cultural anthropological research suggests that sexuality-related behaviors can foster positive outcomes within appropriate organizational contexts. For instance, Lerum (2004)'s ethnographic study revealed that sexual jokes could enhance camaraderie among restaurant staff when occurring within established organizational norms. More recently, Sheppard et al. (2020) demonstrated that when recipients favorably perceive sexuality-related interactions, these experiences can yield positive individual-level outcomes, including stress reduction and tension relief.

However, prior research has primarily focused on the immediate psychological effects of social sexual behavior (SSB), leaving the potential link between SSB and employee innovation largely unexamined. In the era of the 4th Industrial Revolution, innovative behavior is increasingly recognized as a vital contributor to organizational survival and sustainable growth (Anderson et al., 2014; Kahn, 2018). This empirical gap may be partly explained by the cultural sensitivities and regulatory constraints present in specific national contexts. In South Korea, public discourse around sexuality-related behavior has become more restrictive since the 1990s, following prominent incidents such as the 1993 Seoul National University sexual harassment case and the #MeToo movement in 2018. These events heightened public scrutiny and led to the implementation of stringent organizational policies aimed at eliminating any potentially inappropriate conduct. While these policies are well-intentioned, they may also discourage socially constructive interpersonal interactions that are perceived as respectful and pleasant. As Schultz (2003) and Williams et al. (1999) suggest, overly punitive or preventive approaches can suppress natural forms of social engagement, foster hyper-surveillance, and create a climate of interpersonal anxiety. In this context, even benign expressions of social sexual behavior may be perceived as unacceptable, thus eroding opportunities for positive connection and emotional well-being at work.

Jang (2022) introduced the concept of socially attractive behavior as a culturally relevant framework to examine relational dynamics in Korean organizational settings. This type of behavior, when perceived positively by others, may serve to alleviate stress and foster a sense of belonging among employees. Building on this conceptual foundation, the present study investigates how favorable perceptions of SSB contribute to the development of psychological resources and, ultimately, to innovation. Understanding SSB not merely as a risk factor but as a potential psychological and interpersonal asset calls for a more balanced and nuanced approach to sexuality-related behavior in organizations—especially in rapidly evolving workplace cultures that demand creativity, inclusion, and emotional intelligence.

This study makes several unique contributions to the literature. Most notably, it is the first empirical investigation to simultaneously examine two critical boundary conditions—gender and frequency—in the context of workplace SSB. While previous studies have considered these factors independently, their joint examination provides a more nuanced and comprehensive understanding of how SSB operates in organizational settings. This dual-moderator approach allows us to uncover not only who is most affected by SSB (gender differences) but also under what conditions these effects are optimal (frequency threshold).

Building on this novel approach, we examine how favorable perceptions of SSB influence innovative behavior among South Korean private sector employees, pursuing three specific objectives. First, we investigate the direct effects of SSB on both psychological social resources and innovative behavior, examining how these workplace interactions contribute to employee outcomes. Second, drawing on gender role theory and acknowledging that men and women may interpret sexuality-related interactions differently due to biological and sociocultural factors (Gutek, 1985; Williams et al., 1999), we examine gender’s moderating effect on the relationship between SSB and psychological social resources. Third, based on optimal stimulation level theory (Steenkamp and Baumgartner, 1992), which suggests that SSB’s positive effects may diminish or reverse with increasing frequency, we investigate frequency as another critical boundary condition affecting the SSB-psychological resources relationship. Through examining these simultaneous moderating effects, this study not only establishes contextual boundaries for SSB’s effectiveness but also provides organizations with more precise, evidence-based guidelines for managing workplace interactions to enhance innovation while maintaining appropriate interpersonal dynamics.

This integrated examination of both gender and frequency as moderators represents a significant advancement in our understanding of workplace SSB, moving beyond simple main effects to reveal the complex interplay of individual differences and behavioral patterns in shaping organizational outcomes.

## 2 Theoretical background and hypothesis

### 2.1 The concept of social sexual behavior

Social sexual behavior (SSB) in organizations represents interpersonal interactions that contain sexual elements or undertones but are neither harassing nor hostile in nature. Building on this conceptual foundation, our study examines how favorable perceptions of SSB influence innovative behavior and psychological social resources among South Korean private sector employees. We specifically focus on three key aspects: First, we investigate the direct relationships between SSB and both psychological social resources and innovative behavior. Second, drawing on gender role theory (Gutek, 1985; Williams et al., 1999), we examine how gender moderates the relationship between SSB and psychological social resources, acknowledging that men and women may interpret sexuality-related interactions differently due to biological and sociocultural factors. Third, based on optimal stimulation level theory (Steenkamp and Baumgartner, 1992), we investigate how SSB frequency moderates its effectiveness, recognizing that excessive frequency might diminish or reverse its positive effects.

### 2.2 Favorable perceptions of social sexual behavior and social psychological resources

Favorable perceptions of SSB can positively influence the accumulation of psychological social resources among organizational members. To establish this relationship, we draw upon research findings across multiple disciplines, particularly given the nascent state of SSB research.

Cohen and Wills (1985) identified positive interpersonal interactions as a primary mechanism for resource accumulation. Building on this foundation, Sheppard et al. (2020) proposed that individuals who favorably perceive SSB can acquire psychological social resources. This proposition is supported by evidence that positive sexuality-related interactions within organizations can enhance members’ sense of belonging and inclusiveness (Dellinger and Williams, 2002; Erickson, 2010; Giuffre and Williams, 1994; Lerum, 2004).

The positive effects of SSB have been documented across various organizational contexts. For instance, Lerum (2004)'s qualitative study in the service industry revealed that appropriate sexual jokes fostered feelings of freedom and empowerment among employees. Similarly, in the media industry, Dellinger and Williams (2002) found that sexuality-related conversations among female magazine staff members functioned as positive interactions that strengthened interpersonal bonds.

The mechanism through which SSB contributes to resource formation is particularly noteworthy. Being the recipient of SSB implies recognition of one’s attractiveness, potentially enhancing confidence and perceived competence (Hakim, 2010; Loe, 1996). This recognition can translate into informal power (Watkins et al., 2013) and social capital within the organization. Hakim (2010) conceptualized this as ‘erotic capital,’ explaining its role in accumulating psychological social resources.

In this study, psychological social resources are conceptualized through three interrelated dimensions: social confidence, perceived attractiveness, and sense of inclusion. These dimensions reflect affective and relational resources that are most likely to be influenced by favorable perceptions of SSB. Building on the Conservation of Resources (COR) theory (Hobfoll, 1989) and the broaden-and-build theory (Fredrickson, 2001), we posit that SSB functions as a form of interpersonal affirmation that signals appreciation, approachability, and inclusion. These signals contribute to the accumulation of emotional and relational resources, which in turn foster the cognitive and psychological capacity necessary for innovative behavior.

In supervisor-subordinate relationships, which represent crucial organizational interactions, subordinates’ favorable perceptions of their supervisor’s SSB likely contribute to their psychological resource accumulation. Specifically, when subordinates favorably perceive their supervisor’s SSB, they are more likely to accumulate psychological social resources. Therefore, we hypothesize:

*H1: Subordinates' favorable perceptions of their supervisor's social sexual behavior will positively affect their psychological social resources.*

### 2.3 Psychological social resources and innovative behavior

The relationship between psychological social resources and innovative behavior can be explained through Conservation of Resources (COR) theory (Hobfoll, 1989). COR theory posits that individuals are fundamentally motivated to preserve and acquire resources, with resource accumulation promoting individual growth and development. Psychological social resources, including social support, autonomy, and self-esteem, are particularly crucial for individual well-being and growth (Halbesleben et al., 2014; Ten Brummelhuis and Bakker, 2012).

These accumulated psychological social resources can facilitate innovative behavior through several mechanisms. First, abundant psychological resources provide the psychological stability necessary for tackling challenging tasks (Amabile, 1988). Second, enhanced self-esteem and self-efficacy create the psychological latitude needed to propose and experiment with novel ideas (Mumford and Gustafson, 1988). Third, a strong sense of belonging encourages change-initiating behavior beneficial to the organization (Scott and Bruce, 1994).

Empirical studies have consistently demonstrated positive relationships between psychological social resources and key components of innovative behavior, including creativity, problem-solving, and change initiation. Individuals with abundant psychological resources possess the capacity to attempt novel approaches to challenging tasks and can risk failure due to their confidence in eventual success. Moreover, high levels of belongingness and psychological safety within the organization motivate employees to more actively initiate changes and propose innovative ideas. Based on these theoretical foundations, we hypothesize:

*H2: Subordinates' psychological social resources will positively affect their innovative behavior.*

### 2.4 Favorable perceptions of social sexual behavior and innovative behavior

Innovation represents a complex process involving both originality in breaking constraints and practicality in implementing novel ideas (Gebert et al., 2010). Notably, innovative behavior rarely stems from individual creativity alone but requires interaction and collaboration with various organizational members (Amabile et al., 1996).

Leader support and team interaction quality serve as crucial antecedents of innovative behavior (Scott and Bruce, 1994). Janssen (2005) demonstrated that supervisor and colleague support are essential when employees propose and implement innovative ideas, highlighting how positive interactions within social networks facilitate the innovation process.

Subordinates’ favorable perception of their supervisor’s SSB can enhance innovative behavior through several mechanisms. First, it signifies an amicable and supportive supervisor-subordinate relationship, creating an environment where subordinates feel secure taking innovation-related risks. Edmondson (1999) found that employees engage in more innovative attempts when they believe their ideas and potential mistakes will be accepted. Second, since innovation requires breaking from established frameworks, positive supervisor interactions can catalyze subordinates’ creative potential. Third, these interactions contribute to an organizational climate conducive to freely proposing and implementing innovative ideas. Therefore, we hypothesize:

*H3: Subordinates' favorable perception of supervisors' social sexual behavior will positively affect their innovative behavior.*

### 2.5 Moderating effects between social sexual behavior and psychological social resources

A multifaceted research approach is required to deepen our understanding of social sexual behavior, which is still in its early research stages. Particularly, identifying the boundary conditions under which the effects of social sexual behavior manifest is essential for theoretical refinement of this concept. This study focuses on the moderating effect of gender in the process of converting favorable perceptions of social sexual behavior into psychological social resources.

#### 2.5.1 Moderating effect of gender

According to Gender Role Theory, gender role stereotypes and expectations internalized through socialization processes significantly influence individual cognition, emotion, and behavior (Eagly, 1987). While femininity is associated with characteristics such as interpersonal orientation, sensitivity, and cooperativeness, masculinity is linked to traits such as independence, competitiveness, and task orientation (Bem, 1974). These differences can affect how individuals perceive others’ social sexual behavior and acquire resources accordingly. In South Korean organizations shaped by Confucian values and hierarchical leadership structures, gendered experiences may be further amplified. Female employees may interpret favorable SSB from supervisors as a rare and meaningful form of social affirmation or inclusion—especially in male-dominated environments where such relational cues are less frequently directed toward them. This cultural dynamic may enhance women’s receptivity to positively perceived SSB and its psychological impact.

Empirical studies consistently report gender differences in interpersonal sensitivity. Women tend to be more sensitive and attentive to interpersonal cues and interactions compared to men (Briton and Hall, 1995) and show higher receptivity to non-verbal communication and emotional expression (Hall, 1978; McClure, 2000). More notably, women tend to value sociopsychological resources gained through relationships more highly than men. Jordan et al.'s (1991) self-in-relation model emphasizes that women’s sense of self and well-being derive from bonds with others, mutual empathy, and attachment.

These theoretical foundations and empirical evidence suggest that the impact of supervisors’ social sexual behavior on subordinates’ sociopsychological resources may vary by gender. Specifically, women are predicted to accumulate more sociopsychological resources through favorable relationship formation with supervisors compared to men. Therefore, we propose the following hypothesis:

*H4: Gender will moderate the relationship between favorable perception of SSB and psychological social resources, such that the positive effect will be stronger for women than for men.*

#### 2.5.2 Moderating effect of social sexual behavior frequency

According to Conservation of Resources Theory (COR), individuals possess a basic motivation to acquire and maintain valuable resources (Hobfoll, 1989). From this perspective, supervisors’ social sexual behavior can be perceived as a positive resource by organizational members. However, Optimal Stimulation Level Theory suggests that individuals have an optimal level of stimulation they prefer to receive from their environment (Steenkamp and Baumgartner, 1992). According to this theory, when interpersonal stimuli such as social sexual behavior fall below the optimal level, members feel a lack of interaction and desire more stimulation. Conversely, when stimuli exceed the optimal level, members experience psychological fatigue, leading them to avoid stimulation or evaluate its value lower. This phenomenon was confirmed in Frederick and Loewenstein’s (1999) research on hedonic adaptation, and Lian et al. (2017) empirically demonstrated the ‘adaptation’ phenomenon where repeated exposure to specific resources diminishes their effect.

From this theoretical perspective, supervisors’ social sexual behavior initially acts as a positive stimulus that significantly increases members’ sociopsychological resources. However, when its frequency exceeds an individual’s optimal stimulation level, members begin to experience psychological fatigue, consequently evaluating the value of sociopsychological resources provided by social sexual behavior progressively lower. Wilson and Gilbert (2008) explain that this phenomenon relates to the process by which individuals reinterpret recurring experiences as ordinary. Integrating these two theoretical frameworks offers a more dynamic explanation of the observed moderation effect. COR theory accounts for why positively perceived SSB initially functions as a valuable psychological resource by enhancing employees’ sense of inclusion, self-worth, and confidence. In contrast, OST theory explains why the psychological utility of this resource may diminish when the frequency of SSB exceeds an individual’s optimal threshold for interpersonal stimulation. This integrated perspective aligns with our empirical findings: while SSB had a strong positive effect on psychological resources at lower frequencies, this effect weakened and became statistically non-significant at higher frequencies. Rather than reversing, the effect appears to be subject to perceptual saturation or hedonic adaptation, where repeated exposure reduces the behavior’s novelty and psychological impact. These insights underscore the importance of managing SSB frequency in a way that maintains its potential as a constructive resource without overexposure.

These theoretical foundations and empirical studies suggest that as the frequency of social sexual behavior increases, its favorable perception may have a progressively diminishing positive effect on sociopsychological resources. Therefore, we propose the following hypothesis:

*H5: SSB frequency will moderate the relationship between favorable perception of SSB and psychological social resources, such that the positive effect will be weaker when SSB frequency is high.*

**Figure 1:**
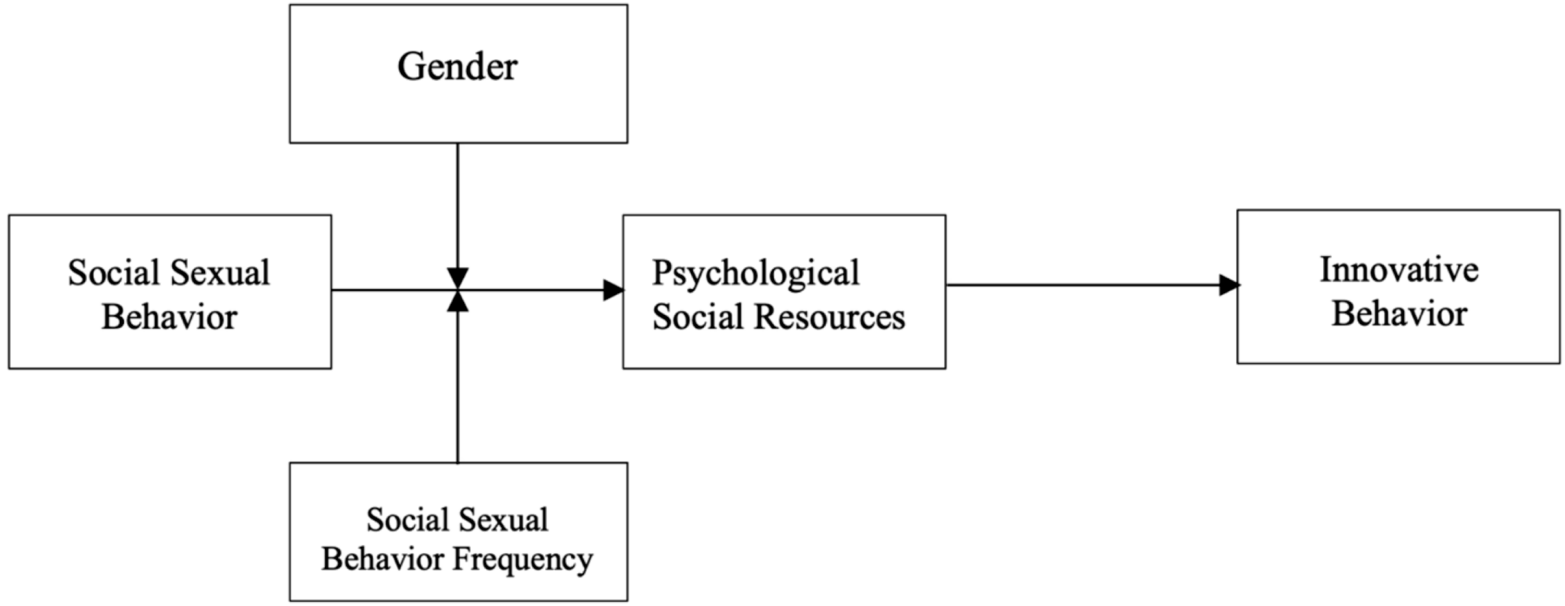
Theoretical model.

According to the above hypotheses, the research model is established as shown in Figure 1.

## 3 Methods

### 3.1 Sample and data collection

Data were collected through a large domestic online survey company in South Korea. The survey targeted office workers who had direct supervisors, and to prevent common method bias, the survey was conducted in two waves with a two-month interval. The first wave garnered responses from 288 participants, and 212 of these respondents completed the second wave survey 2 months later. The final analysis included 212 respondents who completed both surveys (73.6%). Responses from the first and second waves were matched using unique IDs provided by the online survey company. The demographic characteristics of our sample (*n* = 212) showed the following distributions. In terms of gender, the sample was almost evenly distributed with 108 males (50.94%) and 104 females (49.06%). The age distribution showed that most respondents were in their 30s (41.51%) and 40s (32.08%), followed by those in their 50s-60s (39, 18.4%), while those in their 20s represented 8.02% (17) of the sample. Regarding position levels, the sample consisted of staff (18.4%), assistant managers (28.77%), managers (20.28%), deputy general managers (14.15%), general managers (13.68%), and executives or higher (4.72%). Educational background of the respondents showed that the majority held bachelor’s degrees (65.57%), followed by associate degrees (14.62%), master’s degrees (10.85%), high school diplomas or less (8.49%), and doctoral degrees (0.47%). The organizational tenure distribution revealed that the largest group had 5–10 years of experience (27.83%), followed by 1–5 years (22.64%), 10–15 years (18.87%), 15–20 years (13.21%), over 20 years (10.38%), and less than 1 year (7.08%).

### 3.2 Measures

All survey items were measured using a 5-point Likert scale (1 = “strongly disagree” to 5 = “strongly agree”). Social sexual behavior is defined as “interactions between two or more organizational members that include sexual content or metaphors, but without intent to demean others or inflict humiliation or insult, and where the recipient does not perceive the behavior as humiliating or insulting” (Jang, 2022, p.3). Nine items from Sheppard et al. (2020), translated and validated by Jang (2022), were used. The translation and validation process followed a two-stage procedure. First, the original English items were translated into Korean using Brislin’s (1970) back-translation method. Two bilingual experts performed independent forward and backward translations, and a panel of three organizational psychology experts reviewed the translated items for semantic accuracy and cultural appropriateness. Second, the scale was validated in a Korean context through content validity review and factor analysis. A pilot study was conducted to confirm clarity and reliability, and discriminant validity with sexual harassment scales was tested to ensure conceptual distinction between SSB and negative sexual behavior (Jang, 2022). Innovative behavior refers to efforts to seek and share new ideas and develop optimal plans for implementing selected ideas in organizational tasks (Scott and Bruce, 1994). Three items from Schuh et al. (2018) were used to measure this construct. Social psychological resources were measured using five items from Sheppard et al. (2020), assessing confidence, attractiveness, motivation, and sense of belonging.

Two moderating variables were included in this study. Gender was coded dichotomously (1 = male, 2 = female). SSB frequency was measured using a single item asking respondents to report their daily experience of social sexual behavior from their supervisor, rated on a 5-point scale (1 = “never (0 times),” 2 = “rarely (once a day),” 3 = “sometimes (2–3 times a day),” 4 = “often (4–5 times a day),” 5 = “very often (6 or more times a day)”).

Age, education level, and tenure were included as control variables based on prior research (Sheppard et al., 2020; O’Leary-Kelly et al., 2009; Aquino et al., 2014), which suggests that these demographic factors may influence how employees perceive and respond to sexuality-related behaviors. For instance, differences in career stage or educational background can shape individuals’ interpersonal sensitivity and interpretation of social interactions at work.

## 4 Results

### 4.1 Descriptive statistics, correlations, and reliability

To assess the psychometric properties of our measures, we conducted several analyses. Internal consistency reliability was evaluated using Cronbach’s alpha, which yielded satisfactory coefficients for favorable perception of social sexual behavior (*α* = 0.88), social psychological resources (*α* = 0.89), and innovative behavior (*α* = 0.83), all exceeding the conventional threshold of 0.70 (Nunnally and Bernstein, 1994). Exploratory factor analysis (EFA) was conducted using principal component analysis with varimax rotation. All items demonstrated factor loadings exceeding 0.60, with the exception of one social sexual behavior item (Kaiser-Meyer-Olkin measure = 0.83, indicating adequate sampling adequacy). Subsequently, confirmatory factor analysis (CFA) was performed to validate the measurement model.

The three-factor model demonstrated mixed fit: SRMR (0.05) and explanatory power (CD = 0.99) were acceptable, but RMSEA (0.11), CFI (0.89), and TLI (0.88) were slightly below conventional thresholds (Hu and Bentler, 1999). These results may reflect the measurement challenges associated with the emerging construct of social sexual behavior (SSB), particularly within East Asian organizational contexts. Future research should consider scale refinement and broader psychometric validation. To address potential common method bias, we employed Harman’s single-factor test. The unrotated factor solution revealed that the first factor accounted for 28.89% of the total variance, substantially below the 50% threshold, suggesting that common method bias does not pose a significant threat to our results (Podsakoff et al., 2003).

The variables’ means, standard deviations, and correlations were analyzed (see Table 1). The correlations between the variables were largely consistent with theoretical expectations. Specifically, social sexual behavior (SSB) showed a significant positive correlation with psychological social resources (PSR) (*r* = 0.12, *p* < 0.05), and PSR demonstrated a significant positive correlation with innovative behavior (*r* = 0.44, *p* < 0.05). These findings align with previous research suggesting that employees who experience social sexual behavior tend to have enhanced psychological social resources, which in turn contributes to innovative behavior. The moderating variable SSB frequency showed strong positive correlation with social sexual behavior (*r* = 0.54, *p* < 0.01) and psychological social resources (*r* = 0.19, *p* < 0.05), but did not exhibit significant correlation with innovative behavior (*r* = 0.12, n.s.). Gender showed significant negative correlations with SSB (*r* = −0.26, *p* < 0.01), PSR (*r* = −0.18, *p* < 0.05), and innovative behavior (*r* = −0.19, *p* < 0.05), suggesting potential gender differences in the experience and outcomes of workplace social sexual behavior.

**Table 1:** Each variable’s means, standard deviations, correlations, and consistency coefficients.

Variables	Mean	SD	1	2	3	4	5	6	7	8
1. Age	2.64	0.94	1.00							
2. Education level	2.80	0.76	−0.09	-						
3. Tenure	3.40	1.42	0.44*	0.09	-					
4. SSB	2.29	0.77	−0.07	0.06	0.04	-				
5. PSR	3.06	0.77	0.21*	0.19*	0.21*	0.12*	-			
6. IB	3.49	0.71	0.08	0.21*	0.11	0.01*	0.44*	-		
7. gender	1.49	0.50	−0.36*	−0.07	−0.37*	−0.26*	−0.18*	−0.19*	-	
8. SSB frequency	1.89	0.75	−0.01	0.05	−0.01	0.54*	0.19*	0.12	−0.15*	-

*N* = 212, list-wise deletion. SSB = Social sexual behavior, PSR = Psychological social resources IB=Innovative behavior, Gender: male = 1, female = 2, * *p* < 0.05, ** *p* < 0.01, two-tailed tests.

### 4.2 Test of hypotheses

We tested our hypotheses using hierarchical regression analyses (Table 2). Hypothesis 1 predicted that subordinates’ favorable perceptions of their supervisors’ social sexual behavior (SSB) would positively affect their psychological social resources. The results supported this hypothesis, demonstrating a significant positive relationship between SSB and psychological social resources (*β* = 0.12, *p* < 0.05, Model 1). Hypothesis 2 proposed that psychological social resources would positively influence innovative behavior. Our analysis strongly supported this hypothesis, revealing that psychological resources significantly enhanced innovative behavior (*β* = 0.41, *p* < 0.01, Model 2). This relationship explained a substantial portion of variance in innovative behavior (ΔR^2^ = 0.15), suggesting that psychological resources play a crucial role in fostering employee innovation. Hypothesis 3, which predicted a direct positive relationship between SSB and innovative behavior, received marginal support (*β* = 0.02, *p* < 0.05, Model 1). This relatively weak direct effect, compared to the stronger indirect path through psychological resources, suggests that SSB’s influence on innovation may primarily operate through its impact on psychological resources. The moderation hypotheses revealed particularly interesting patterns. Hypothesis 4, which predicted gender differences in the SSB-psychological resources relationship, was strongly supported. The significant interaction term (*β* = 0.53, *p* < 0.05, Model 3) indicated that women experienced more pronounced psychological benefits from favorable SSB perceptions compared to men. This finding was further substantiated by simple slope analyses, which revealed a stronger positive relationship for female employees (*β* = 0.65, *p* < 0.01) compared to male employees (*β* = 0.12, *p* < 0.05). Hypothesis 5, concerning the moderating role of SSB frequency, was also supported and revealed an important boundary condition. While SSB frequency initially showed a positive main effect (*β* = 0.17, *p* < 0.05, Model 2), the interaction term (*β* = −0.69, *p* < 0.05, Model 3) indicated that excessive frequency could diminish the positive effects of SSB. Simple slope analyses clarified this relationship: the positive effect of SSB on psychological resources was stronger at lower frequencies (*β* = 0.57, *p* < 0.01) but became non-significant at higher frequencies (*β* = −0.12, n.s.). This finding suggests an optimal level of SSB frequency beyond which additional interactions may not yield further psychological benefits. Among control variables, both age (β = 0.20, *p* < 0.01) and education (*β* = 0.20, *p* < 0.01) demonstrated significant positive effects on psychological social resources, while education maintained its positive influence on innovative behavior (*β* = 0.12, *p* < 0.05) in the final model. The overall model explaining psychological social resources showed good explanatory power (*R*^2^ = 0.15, *F* = 6.09, *p* < 0.01), supporting the robustness of our findings.

**Table 2:** Results of the hierarchical multiple regression analysis of the study variable’s effects on task performance and psychological capital; standardized coefficients (*n* = 212).

Variables	Psychological social resources			Innovative Behavior		
	Model 1	Model 2	Model 3	Model 4	Model 5	Model 6
Control variables						
Age	0.20**	0.18**	0.19	0.07	0.01	−0.01
Education	0.20**	0.20**	0.19	0.21**	0.12*	0.12*
Tenure	0.09	0.11	0.09	0.06	0.02	0.02
Main effects						
Social Sexual Behavior	0.12*	0.02*	0.40	0.02*		0.07
Gender^1^						
Frequency^2^						
Two-way interactions						
SSB × Gender	0.53*					
SSB × SSB Frequency	−0.69*					−0.08*
Mediator						
Psychological Resources					0.41**	0.42**
F	6.61**	6.37**	6.09**	3.08*	13.70***	11.21***
R^2^	0.11	0.13	0.15	0.06	0.21	0.21
Adjusted R^2^	0.04	0.11	0.13	0.04	0.19	0.19
R^2^ change	1.50	1.42	1.47	1.42	1.38	1.33

*N* = 212. Standardized regression coefficients are reported. SSB = Social Sexual Behavior. ^1^Gender was coded as 1 = male, 2 = female, ^2^Frequency was measured on a 5-point scale (1 = never to 5 = very often), *p < 0.05; **p < 0.01; ***p < 0.001 (two-tailed tests), 95% confidence intervals are reported in parentheses.

Additional t-tests were conducted using the method suggested by Aiken et al. (1991) to closely examine the interaction effects. The significance of the correlation coefficients (simple slope; Aiken et al., 1991) was examined after calculating values at high (+1 SD) and low (−1 SD) levels of the moderating variables.

**Figure 2:**
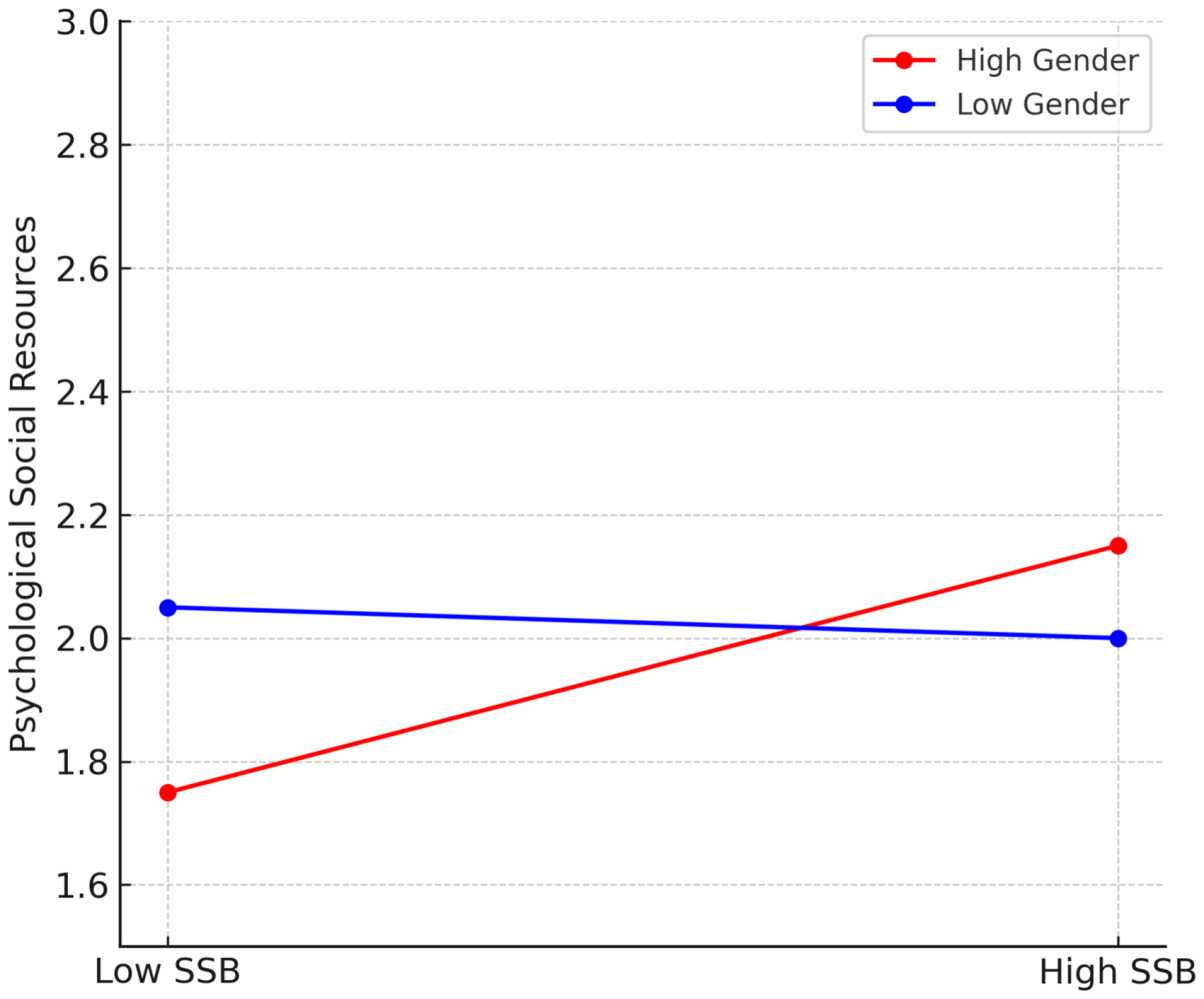
Moderating effect of gender on the relationship between SSB (social sexual behavior) and psychological social resources. The positive effect of SSB on psychological resources is stronger among female employees compared to male employees.

For gender moderation, the simple slope analysis revealed that the relationship between SSB and psychological social resources was stronger for female employees (*b* = 0.65, *p* < 0.01) compared to male employees (*b* = 0.12, *p* < 0.05), supporting Hypothesis 4 (Figure 2).

**Figure 3:**
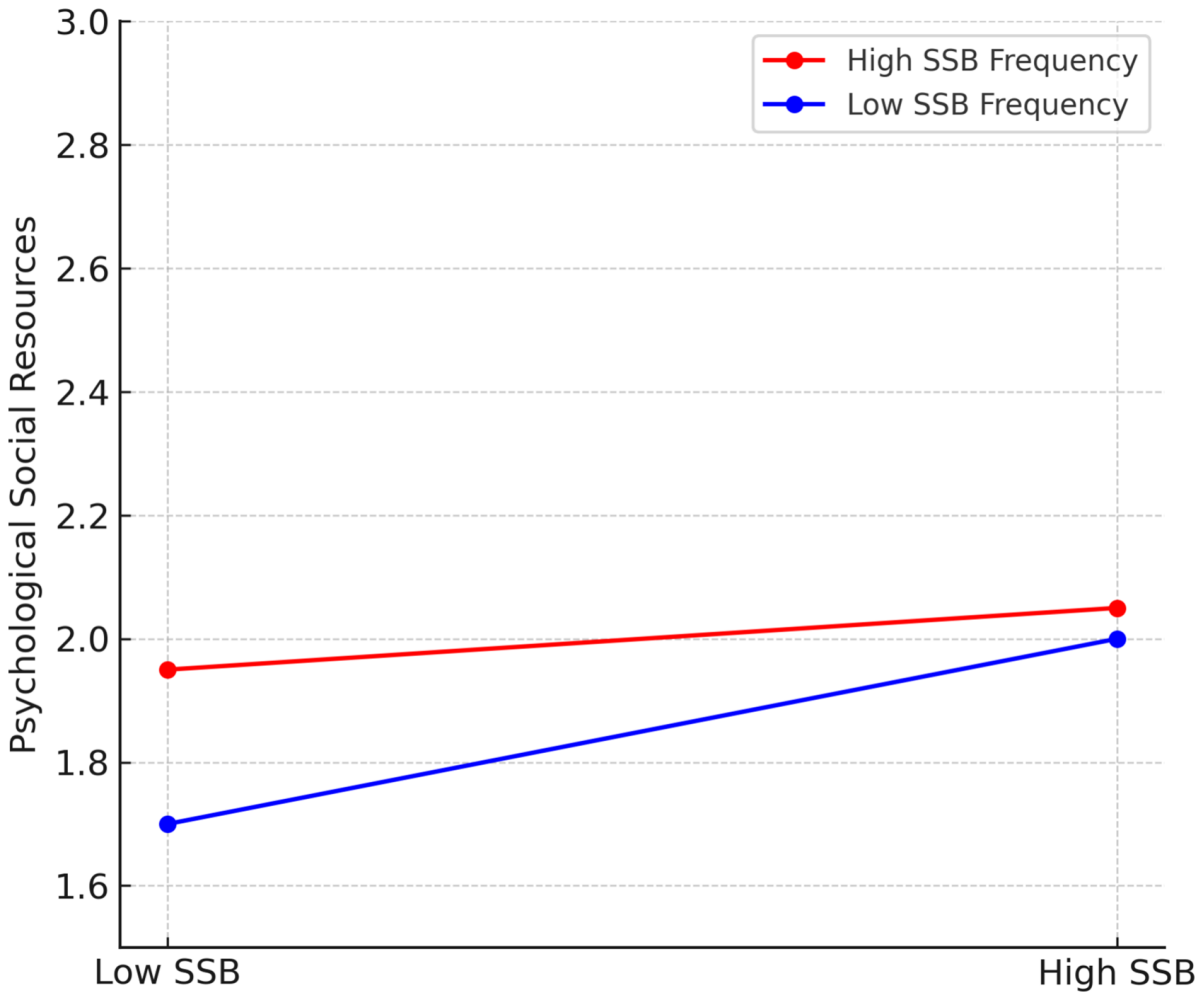
Moderating effect of gender on the relationship between SSB (social sexual behavior) frequency and psychological social resources. The effect of SSB is stronger at low levels of frequency and becomes non-significant at high frequency levels.

For frequency moderation, the regression coefficients and significance levels varied when SSB frequency was high (*b* = −0.12, n.s.) versus low (*b* = 0.57, *p* < 0.01). This demonstrates that higher frequency of SSB weakens its positive influence on psychological social resources, supporting Hypothesis 5 (Figure 3).

## 5 Conclusion

This study holds significant academic value in empirically identifying the novel concept of ‘social sexual behavior’ within the organizational context of South Korea. Discussions on social sexual behavior, an area still lacking in research, remain in the early stages without a rich theoretical and empirical foundation. Recognizing the limitations of quantitative research methodologies predominantly used in organizational behavior, this study aims to explore the mechanisms and influences of social sexual behavior by borrowing qualitative research findings accumulated in adjacent academic fields such as anthropology. This approach can contribute to expanding the horizon of research on social sexual behavior.

### 5.1 Summary of findings

This study aimed to examine how subordinates’ favorable perception of supervisors’ social sexual behavior influences innovative behavior, focusing on the mediating role of sociopsychological resources and moderating effects. Data were collected through two-wave online surveys from 212 office workers in South Korea who had direct supervisors.

Our analysis revealed three main effects. First, subordinates’ favorable perception of supervisors’ social sexual behavior positively influenced their sociopsychological resources. Second, subordinates’ sociopsychological resources positively affected their innovative behavior. Third, favorable perception of supervisors’ social sexual behavior had a direct positive effect on innovative behavior.

Particularly noteworthy are two moderating effects. First, gender moderated the relationship between favorable perception and sociopsychological resources, with women showing stronger positive effects than men when favorably perceiving their supervisor’s social sexual behavior. This finding requires careful interpretation. While it might appear to suggest that men maintain consistent levels of psychological resources regardless of their supervisor’s social sexual behavior, while women’s psychological resources are heavily influenced by it,” such an interpretation would be biased and misaligned with our research intent. Rather, this finding should be understood as reflecting different experiences and perceptions between genders within organizational interactions. However, it is important to clarify that these observed gender differences are not meant to imply biologically inherent traits or normative expectations. Rather, they likely reflect the interaction of gender with contextual and cultural variables—such as communication norms, organizational hierarchies, and perceptions of power. We strongly caution against overgeneralizing these findings and encourage future research to adopt an intersectional perspective when interpreting gendered responses to SSB in professional settings.

Considering the measurement items of social sexual behavior, women’s heightened sensitivity to such behaviors may be related to the nature of organizational interactions. For example, compliments about appearance or conversations about romantic relationships may have a greater impact on female members’ sociopsychological resource formation (Berdahl and Aquino, 2009).

Second, we found a moderating effect of frequency. Specifically, the positive impact of favorable perception on sociopsychological resources was strongest at low to moderate levels of social sexual behavior but diminished at high frequencies. This suggests the existence of an “optimal frequency” for sex-related social sexual behavior, highlighting both the need for strategic implementation and recognition of its limitations in organizational settings.

This study has significant academic implications as it empirically validates the concept of social sexual behavior in South Korean organizational contexts. While previous domestic organizational behavior research has primarily focused on negative aspects such as sexual harassment and workplace bullying, our study demonstrates that healthy and favorable sexual interactions can exist and significantly influence organizational members’ attitudes and behaviors. By broadening the horizon of social sexual behavior research, which is still in its early stages both domestically and internationally, this study provides an empirical foundation for future multifaceted exploration of this phenomenon. Our findings contribute to understanding gender-based differences in organizational experiences and perceptions, potentially aiding in the development of better organizational cultures.

### 5.2 Practical implications

This study provides the following theoretical implications:

First, this study is significant in that it sheds new light on sex-related behaviors, which were mainly dealt with from a negative perspective in existing organizational behavior research, through the concept of social sexual behavior, and empirically verified the impact of favorable perceptions of such behavior on members’ behaviors and attitudes. This contributes to deepening the understanding of social sexual behavior in organizations, which is still in its early stages.

Second, this study established a theoretical foundation for the concept by explaining the operating mechanism of social sexual behavior based on Conservation of Resources Theory. Specifically, it revealed that social sexual behavior contributes to preserving and enhancing individual psychological resources. This is significant in that it applies existing organizational behavior theories in a new context.

Third, by discussing and empirically demonstrating the moderating effect of gender from the perspective of gender role theory, this study provides a theoretical explanation of how social sexual behavior is interpreted differently and impacts differently according to gender within organizations. In particular, the finding that the positive effectiveness of favorable perceptions of supervisor’s social sexual behavior leading to social psychological resources is strengthened among female members provides new insights into gender-related social interactions within organizations.

Fourth, by empirically verifying the moderating effect of social sexual behavior frequency, this study revealed the complexity and multifaceted nature of this concept. Based on Conservation of Resources Theory (Hobfoll, 1989) and the ‘adaptation’ phenomenon by Lian et al. (2017), it was confirmed that the positive effects of social sexual behavior may decrease as its frequency increases. This suggests that there may be an ‘optimal frequency’ of social sexual behavior, and this finding shows that social sexual behavior is not simply positive or negative but can have various effects depending on its frequency and context. This makes an important theoretical contribution to understanding the complexity of social interactions within organizations.

The practical implications of this study are as follows:

First, there is a need to shift prevailing perceptions of social sexual behavior (SSB) in the workplace. While such behaviors have traditionally been viewed uniformly through a negative lens, our findings suggest that positively perceived SSB can function as a healthy form of interpersonal communication that supports the development of innovative behavior. Organizations should work to recognize and understand the constructive potential of such interactions, while maintaining clear ethical standards.

Second, the perception and interpretation of SSB by subordinates are more influential than the behavior itself. When subordinates interpret their supervisor’s behavior positively, it fosters key psychological social resources such as emotional stability, a sense of belonging, and self-esteem—all of which contribute to enhanced innovation. Organizations should foster environments that help employees interpret interpersonal cues within safe and respectful boundaries.

Third, gender-based differences in how SSB is perceived and internalized must be considered. The study found that female employees derived stronger psychological benefits from positively perceived SSB. This suggests that organizational guidelines should account for gender-specific experiences and promote interactions that are inclusive, respectful, and sensitive to interpersonal diversity.

Fourth, managing the frequency of SSB is equally critical. Our results indicate that while moderate levels of SSB may be positively received, excessive frequency may reduce its effectiveness and even generate discomfort. Importantly, this “optimal frequency” is not a normative recommendation, but a descriptive finding based on perceptual patterns observed in this study. Organizations must interpret this result with caution, ensuring that any application is aligned with ethical principles, mutual respect, and organizational policy. Maintaining appropriate levels of interpersonal behavior can help foster a psychologically safe workplace and promote sustainable innovation.

### 5.3 Limitations and future research

This study has several limitations that suggest directions for future research. First, methodological constraints of self-reported measures may introduce common method bias and social desirability concerns, particularly given the sensitive nature of SSB. Future research should incorporate multi-source evaluations and objective indicators. To address this limitation, future studies should consider employing methodological triangulation—such as multi-source assessments, behavioral observation, or dyadic supervisor-subordinate designs—to enhance objectivity and better capture the relational dynamics of SSB. Second, while our two-wave design provides temporal separation, future studies should employ more sophisticated longitudinal designs to better capture the dynamic nature of SSB effects. Third, our study did not fully address multilevel factors affecting SSB effectiveness; future research should consider individual, organizational, and cultural contexts through multilevel analysis. Fourth, we did not account for potential discrepancies between supervisor intentions and subordinate perceptions of SSB, suggesting future studies should examine both perspectives. Finally, cross-cultural comparisons would enhance understanding of how cultural contexts influence SSB interpretation and effects.

## Data Availability

The original contributions presented in the study are included in the article/supplementary material, further inquiries can be directed to the corresponding author.
